# Epidermal barrier: Adverse and beneficial changes induced by ultraviolet B irradiation depending on the exposure dose and time (Review)

**DOI:** 10.3892/etm.2013.1175

**Published:** 2013-06-21

**Authors:** FELICIA PERMATASARI, BINGRONG ZHOU, DAN LUO

**Affiliations:** Department of Dermatology, The First Affiliated Hospital of Nanjing Medical University, Nanjing, Jiangsu 210029, P.R. China

**Keywords:** epidermal barrier, ultraviolet B, suberythemal dose, covalently bound ceramides, calcium gradient, vitamin D_3_

## Abstract

Exposure of the skin to ultraviolet (UV) radiation induces various harmful effects in the tissues, particularly disruption of the epidermal barrier. However, ultraviolet B (UVB) irradiation has been applied in the treatment of atopic dermatitis, a skin disease in which the epidermal barrier is defective. We reviewed the homeostasis of the epidermal barrier and several studies investigating the adverse and beneficial effects caused by different doses of UVB irradiation in the epidermal barrier. It may be concluded that, despite the harmful effects of UVB irradiation on the skin, UVB irradiation is able to exert beneficial effects in the epidermal barrier when administered in suberythemal doses and over a relatively short period of time, with no clinically evident inflammation or barrier disruption. This may be a useful therapeutic strategy for the use of UVB irradiation in the treatment of skin diseases with a disrupted epidermal barrier, such as atopic dermatitis, while reducing or avoiding the side-effects.

## Contents

1. Introduction
2. Homeostasis of the epidermal barrier
3. Adverse effects induced by UVB in the epidermal barrier
4. Reduced levels of covalently bound Cer
5. Disrupted multilamellar structures in the intercellular space of the SC
6. Increased GlcCer levels
7. Disrupted epidermal calcium gradient
8. Beneficial effects induced by UVB in the epidermal barrier
9. Conclusions

## 1. Introduction

The epidermal barrier is important for maintaining the homeostasis of the skin. The skin is constantly exposed to ultraviolet A (UVA) and ultraviolet B (UVB) irradiation while ultraviolet C radiation is absorbed by the ozone layer. Due to their different wavelength ranges, UVA and UVB act at two different levels of the skin. UVA predominantly affects the dermis and the DNA, whereas UVB affects the epidermis (1–3). UV irradiation of the skin is known to induce disruption of the epidermal barrier (4–7). However, UVB irradiation has been used for the treatment of atopic dermatitis, a skin disease involving a defective epidermal barrier (8–10). We reviewed the homeostasis of the epidermal barrier and the previous studies investigating the adverse and beneficial effects of UVB irradiation in the epidermal barrier, with the aim of understanding the potential therapeutic strategy of using UVB irradiation in the treatment of skin diseases with a disrupted epidermal barrier.

## 2. Homeostasis of the epidermal barrier

The skin barrier properties are primarily localized in the outer epidermal layer, the stratum corneum (SC) (11). The SC consists of corneocytes surrounded by a neutral lipid-enriched extracellular matrix. The mechanical strength of the skin is provided by the corneocytes, which are encased by a cornified cell envelope (CE) (12). The hydrophobic extracellular lipid matrix provides a barrier against the movement of water and electrolytes (11). Thus, the SC is involved in the regulation of water release from the organism and into the atmosphere, known as transepidermal water loss (TEWL) (13). TEWL is used as an indicator of the functional integrity of the SC (14,15).

**Figure 1.**
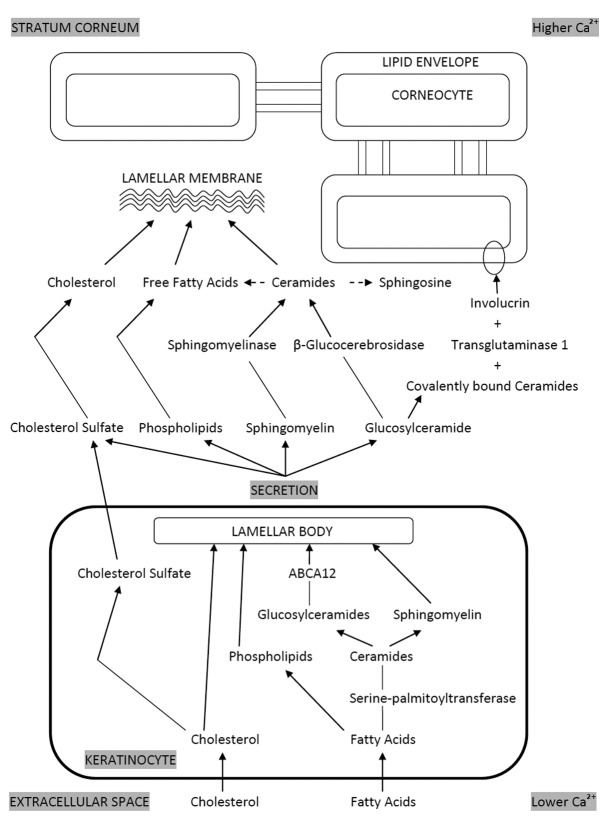
Pathways for the formation of the extracellular lamellar lipid membranes and the lipid envelope.

Lamellar bodies (LBs) are abundantly located in the differentiated keratinocytes, particularly in the stratum granulosum (16). LBs are responsible for supplying the lipids for the lipid envelope of the CE and the extracellular lipid matrix (Fig. 1) (16,17). LBs contain lipid precursors and numerous enzymes, including lipid hydrolases and proteases (16). It is considered that the incorporation of the lipid hydrolases and proteases into LBs requires the prior or concurrent delivery of lipids to the LBs (18). Thus, if lipids are deficient or lipid synthesis is disrupted, the enzymes that are characteristically found in LBs are not transported from the Golgi to the LBs (18). The lipids that constitute the extracellular matrix comprise 15% fatty acids, 25% cholesterol and 50% ceramides (Cer) (16). The relative quantities of these three key lipids are important for the formation of LBs. An excess or deficiency of a particular lipid may disturb LB formation (12).

In the normal human and murine epidermis, the extracellular calcium (Ca^2+^) content is low in the basal and spinous layers, with a gradual increase from the inner to the outer layers and a maximal concentration within the outer stratum granulosum (19–21). The change in calcium concentration appears to be the primary signal inducing LB secretion (12). Following secretion from the granular cells into the intercellular space, these LB-derived lipids are further metabolized in the SC extracellular spaces by enzymes that are cosecreted in the LBs (16,17,22–24). Specifically, β-glucocerebrosidase (β-GlcCerase) converts glucosylceramides (GlcCer) into Cer (25,26).

A minor but important component of the LB-derived lipids are acylglucosylceramides, of which approximately two-thirds are converted to ω-hydroxyceramides that become covalently attached by ester linkages to the CE peptides (27–30). ω-hydroxyceramides are the predominant lipid species of the corneocyte lipid envelope in the epidermis (31). These contain linoleic esters, which are linked by the action of transglutaminase-1 (TGase 1) to glutamine and to glutamate residues of a number of CE structural proteins, most notably involucrin (30,32). These covalently bound Cer contribute a hydrophobic surface to the corneocyte that has important consequences for water barrier function by interactions with, and perhaps organization of, intercellular lipids (33).

## 3. Adverse effects induced by UVB in the epidermal barrier

Excessive exposure to UV radiation may lead to skin cancer and premature aging, and UVB is the most effective waveband at causing these changes (34–36). Exposure of the skin to UVB radiation may induce changes in the epidermal barrier (37).

## 4. Reduced levels of covalently bound Cer

Studies have demonstrated that the levels of covalently bound Cer are significantly reduced in parallel with significant increases in TEWL following irradiation with a single UVB dose of 75 or 160 mJ/cm^2^, and following continuous UVB irradiation [40 mJ/cm^2^ or 0.5 × the minimal erythemal dose (MED)/day for 14 days] in hairless mice and rats (38,39). This suggests a close correlation between the alteration of covalently bound Cer and the UVB-induced perturbation of the skin barrier.

It has been observed that the level of involucrin did not change following a single UVB irradiation at a dose of 50 mJ/cm^2^; however, the expression of TGase 1 mRNA was shown to be significantly downregulated (38,40). Hirao *et al* (41) revealed that a reduction in the binding of ω-hydroxyceramides to involucrin was elicited by UVB irradiation (41). Takagi *et al* (38) suggested that the UVB-induced downregulation of TGase 1 may have been responsible for the observed reduction in the level of covalently bound Cer.

A study in which hairless mice were irradiated with a single 75 mJ/cm^2^ dose of UVB radiation demonstrated that a reduction in the level of covalently bound Cer occurred in parallel with the time when the thickness of the epidermis was significantly increased (38). Treatments known to induce epidermal hyperplasia, such as tape stripping or sodium dodecylsulfate (SDS) treatment, led to significant reductions in the levels of covalently bound Cer, whereas the levels of non-bound Cer remained unchanged (38). This suggests that the decreased level of bound Cer is highly correlated with epidermal hyperplasia (38).

## 5. Disrupted multilamellar structures in the intercellular space of the SC

LBs are secreted from the outer stratum granulosum cells (12). Acute disruption of the permeability barrier initiates a homeostatic repair response, including the rapid secretion (within minutes) of the contents of the LBs (50–80% of pre-existing LBs are secreted) (42–44). A number of studies have demonstrated the suppression of LB release to the space between the SC and granulosa, and the retention of the LBs in the SC, following continuous UVB irradiation daily at a dose of 40 mJ/cm^2^ or 0.5 × MED/day for 14–15 days on hairless rats (39,45). This may have contributed to the resulting disrupted multilamellar structures in the SC (folding, defects or absent). Bound Cer are considered to be formed by the fusion of LBs with the cell membrane of granulosum cells, followed by release into the space between the SC and granulosa (17). Meguro *et al* (39) suggested that the suppression of the release of the LBs into the space between the SC and the granulosa may have resulted in the reduction in covalently bound Cer in the SC following UVB irradiation (39).

A number of studies have shown that bound lipids may act as templates in the formation process of multilamellar structures by LBs released into the space between the SC and granulosa, and may be important in the maintenance of the multilamellar structures by connecting corneocytes to the multilamellar layer, acting as connectors between corneocytes or conferring resistance to proteinases derived from bacteria (17,46,47). Several studies have demonstrated the importance of bound lipid for the formation of multilamellar structures between corneocytes (39,48). In addition, certain studies have demonstrated the disruption of multilamellar structures together with decreased levels of covalently bound Cer following UVB irradiation (38,39). Thus, the reduced levels of covalently bound Cer may contribute to the disruption of multilamellar structures. Covalently bound Cer and the formation of multilamellar structures by LB release may contribute reciprocally to enable normal function, while alterations induced by UVB irradiation affect the normal regulation of the two factors.

## 6. Increased GlcCer levels

Tagaki *et al* (49) observed markedly suppressed epidermal β-GlcCerase activity with a significantly increased expression of β-GlcCerase mRNA, followed immediately by the accumulation of GlcCer in the SC of hairless mice, one day subsequent to a single UVB irradiation dose of 70 mJ/cm^2^(49). Under normal conditions, β-GlcCerase exists at levels sufficient to convert all GlcCer secreted from lamellar granules into Cer, leading to complete loss of GlcCer in the SC. The accumulation of GlcCer in the SC resulting from the inhibition of β-GlcCerase activity leads, in turn, to barrier perturbation, concomitant with the abnormal lamellar integrity (26,49,50).

It has been demonstrated that epidermal hyperplasia may be induced by elevated GlcCer levels (51); thus, the accumulation of GlcCer in the epidermis, as a result of the downregulation of β-GlcCerase, may be the cause of the hyperproliferation induced by UVB irradiation (49).

Decreased β-GlcCerase activity may occur due to damage to the enzyme itself. As β-GlcCerase is predominantly localized in the outer epidermis (52), its activity may be susceptible to UVB irradiation, resulting in an attenuated protein-generating capacity at specific cellular levels of the epidermis (49).

Between 70 and 80% of Cer are derived from GlcCer through the action of β-GlcCerase (53,54). Decreased β-GlcCerase activity, induced by UVB, gives rise to the significant accumulation of GlcCer in the SC; however, it has been observed that this is not accompanied by a significant reduction in the Cer level in the SC (49). Tagaki *et al* (49) demonstrated that the increasing level of GlcCer in the SC following UVB irradiation corresponded to <10% of the total Cer in the SC. It was suggested that β-GlcCerase activity exists below the SC at a level sufficient to convert more than the control level of GLcCer (49).

## 7. Disrupted epidermal calcium gradient

Jiang *et al* (55) revealed the appearance of large clumps of calcium precipitates in the extracellular spaces of the lower nucleated layers of the epidermis of hairless mice 48 h subsequent to UVB irradiation at a dose of 0.15 J/cm^2^ (equivalent to 7.5 × MED) (55). The normal calcium gradient was altered, with higher extracellular calcium levels within the lower layers of the epidermis (55). The most notable feature was observed 96 h subsequent to UVB irradiation, when the TEWL reached the highest level. Immediately following barrier disruption, the increased water movement through the compromised SC carried calcium outward toward the skin surface, resulting in a reduction in the calcium concentration surrounding the stratum granulosum cells (20,56,57). A gradual return to a normal calcium distribution level was observed in parallel with the gradual return to a normal TEWL level (55), indicating that barrier recovery occurs in parallel with the restoration of the calcium gradient in the epidermis (20). This shows that a marked correlation exists between the altered calcium gradient and the disrupted epidermal barrier.

If the reduction in calcium levels is prevented by the provision of exogenous calcium, LB secretion does not occur and permeability barrier repair is not initiated (20,56–58). Conversely, if the calcium levels surrounding the stratum granulosum cells are decreased without disrupting the permeability barrier by either iontophoresis or sonophoresis, LB secretion is stimulated (58,59). This shows that the calcium gradient in the epidermis is closely linked to the presence of a normal permeability barrier.

Several studies have investigated the UVB-induced abnormal lamellar membrane structures in the SC interstices (38,39,60). Defective lamellar multilayers have also been observed in the presence of an altered calcium gradient following UVB irradiation (55). The altered calcium gradient following UVB irradiation may affect the secretion of LBs (20,56,57). This may account for the suppression of LB release to the space between the SC and granulosa, and the retention of LBs in the SC, following the continuous UVB irradiation of hairless rats daily at a dose of 40 mJ/cm^2^ or 0.5 × MED/day for 14–15 days (39,45). This may contribute to the observed abnormal lamellar membrane structures in the SC following UVB irradiation.

Abundant evidence supports the hypothesis that extra-cellular calcium regulates the progression of mammalian epidermal differentiation. In human and murine keratinocytes, low calcium concentrations stimulate proliferation, while high calcium concentrations inhibit proliferation and enhance differentiation (61). Moreover, it has been demonstrated that UVB irradiation induces a variety of cutaneous responses, including the induction of epidermal hyperplasia (55). Thus, it is conceivable that a critical level of cytosolic calcium is required for the initiation of the differentiation events. Therefore, the increases in the extracellular and cytosolic calcium levels are associated with the changes in the epidermal proliferation and/or differentiation process (55).

A study observed that following an increase in the calcium concentration, the TGase 1 enzyme activated glutamine residues (Gln^107^, Gln^118^, Gln^122^, Gln^133^ and Gln^496^) of involucrin, with high specificity (32). Since cornified envelopes are present in the outer epidermis where the calcium concentration is normally higher, TGase 1 functions well in such a high calcium concentration. Therefore, the disruption of the epidermal calcium gradient caused by UVB irradiation may further downregulate TGase 1 enzyme activity, subsequently leading to the disruption of the formation of covalently bound Cer.

## 8. Beneficial effects induced by UVB in the epidermal barrier

Although narrowband UVB or UVA phototherapy is a mainstay of treatment, natural and artificial UVB irradiation is frequently employed in the treatment of atopic dermatitis (9,10). Different skin types are affected differently by the same level of UVB exposure. The MED of a fair-skinned (Fitzpatrick type I) person is 10–25 mJ/cm^2^(62,63), while individuals with darker skin have a higher MED (64). Therefore, in UVB treatment, the skin type of the patient also determines the treatment dose. The exposure dose is an important factor in determining the effects of UVB exposure. UV-induced DNA damage and barrier disruption increase linearly with increasing dosage (7).

High doses of UVB, such as a dose 4-fold higher than the MED, are known to exert detrimental effects on permeability barrier function (37,38,60). By contrast, low-dose UV phototherapy has been demonstrated to be useful for the treatment of a variety of skin disorders, including psoriasis and atopic dermatitis (9,10,65). An erythemal dose (≥1 × MED) may impair DNA repair mechanisms and lead to cell elimination via apoptosis (66,67). A study of hairless mice irradiated with a single dose of ∼1 × MED UVB (75 mJ/cm^2^) showed significant disruption of the barrier (38). A study of human subjects irradiated with 0.7 × MED UVB for 10 consecutive days revealed an unaltered expression of p53, a protein which is able to activate DNA repair proteins when DNA has sustained damage. This indicated that the repair systems were activated (67).

A study on hairless mice exposed to 0.5 × MED UVB irradiation (40 mJ/cm^2^) daily for 14 days demonstrated a significant reduction in the levels of covalently bound Cer and disrupted multilamellar structures (39). By contrast, a separate study concerning hairless mice irradiated with the same dose of UVB daily for 3 days demonstrated no clinically evident inflammation or barrier disruption (68). A dose of 0.5 × MED UVB irradiation for 3 days, prior to tape-stripping, resulted in significantly accelerated barrier recovery rates, implying that repeated, short-term exposure to low-dose UVB significantly accelerates the kinetics of barrier recovery following acute insults (68).

The irradiation of hairless mice with a 0.5 × MED dose of UVB for 3 days demonstrated the positive effects of UVB on the epidermis, which, at least in part, were mediated by cutaneous vitamin D_3_ activation (68). In parallel with the upregulation of the cutaneous vitamin D_3_ system, there was an increase in the mRNA levels for the epidermal lipid synthetic enzymes, HMG-CoA, fatty acid synthase (FAS) and serine palmitoyl transferase (SPT) (68). There was also an upregulation of barrier-linked antimicrobial peptides (AMPs; LL-37 and hBD2) in the outer epidermis, which is considered to be mediated by the cutaneous production of 1,25(OH)_2_D_3_, the most active form of vitamin D_3_(111). Increases in the expression of involucrin and filaggrin were also observed, without the concurrent development of epidermal hyperplasia, implying that UVB may also regulate epidermal differentiation (68).

1,25(OH)_2_D_3_ has been demonstrated to increase the expression of a number of major epidermal differentiation proteins, including involucrin, loricrin, filaggrin and transglutaminase, as well as to stimulate cornified envelope formation (68). A suberythemal dose of UVB exposure is normally enough to generate the synthesis of sufficient vitamin D_3_ to impact downstream events in the epidermis (68). Another study on cultured human keratinocytes showed that irradiation with a single 23 mJ/cm^2^ dose of UVB upregulated SPT activity, leading to increased sphingolipid synthesis (69).

## 9. Conclusions

In addition to TEWL acting as an indicator of the functional integrity of the SC, alterations in covalently bound Cer and the epidermal calcium gradient are closely associated with the disruption of the epidermal barrier.

A single high dose of UVB irradiation and suberythemal doses of UVB irradiation for 14 days have been demonstrated to exert negative effects on the epidermal barrier, leading to barrier disruption. By contrast, a low dose of UVB irradiation and suberythemal doses of UVB irradiation for 3 days have been demonstrated to exert positive effects on the epidermal barrier, without clinically evident inflammation or barrier disruption.

The present review therefore shows that, despite the known harmful effects, UVB irradiation may exert positive effects in the epidermal barrier when administered in low doses and over a relatively short period. This may be a useful therapeutic strategy for the use of UVB irradiation in the treatment of skin diseases with a disrupted epidermal barrier, such as atopic dermatitis, while reducing or avoiding the possible side effects. Further studies are required to determine the efficacy of low doses of UVB irradiation on the skin of patients with atopic dermatitis.
